# PW01-036 – Renal replacement therapy in patients with FMF

**DOI:** 10.1186/1546-0096-11-S1-A89

**Published:** 2013-11-08

**Authors:** M Papazyan, M Voskanyan, H Nazaryan, S Babloyan, A Sarkissian

**Affiliations:** 1 Nephrology, "Arabkir" Medical Centre, Yerevan, Armenia; 2 Paediatric Surgery & Urology, "Arabkir" Medical Centre, Yerevan, Armenia

## Introduction

Renal amyloidosis (RA) of Familial Mediterranean Fever (FMF) – although largely preventable –still is a major health problem in Armenia and an important cause of death.

## Objectives

The **aim** of the study is to investigate the long-term outcome of patients with renal amyloidosis on RRT.

## Methods

From January 2002 till September 2012 279 patients were admitted to our centre for Renal Replacement Therapy (RRT), of whom 40 (14.3%) had RA of FMF. Their mean age was 31.4±12.7 (range 12.6–52.9), 60% were males. Mean duration of hemodialysis (HD) was 1.6±1.7 years (range 0.1-6.0).

## Results

**Hemodialysis**: Of the 28 patients not undergoing renal transplantation (Tx) in Armenia, 9 died (systemic amyloidosis - 5, heart attack - 2, stroke -2); 15 moved to another country and 4 remained on dialysis. Over half of the 25 pts with minimum observation period of 6 mo were resistant to EPO. One third died, mainly due to cardiovascular complications and systemic amyloidosis.

**Living related donor Tx** was done after 8 mo (median) of HD in 12 patients aged 38 ± 11.6 years, i.e. 12.5% of all Tx (96) done at the same period. In addition to standard immunosuppression all received low dose colchicine (0.6-1.2 mg/day). Main complications were rejection (8), delayed graft function for tubular necrosis (2), lymphocele (2), CMV disease (2) and tuberculosis (1). Additional problems included diarrhea (colchicine, MMF, generalized amyloidosis; 9) and severe neuropathy due to interaction of cyclosporine with colchicine (1). Interaction of colchicine with CNI/MMF (neuropathy,diarrhea) required reduction of immunosuppression in some patients, resulting in higher rejection rate. One patient died of generalized amyloidosis and 1 kidney was lost after reduction of immunosuppression due to tuberculosis. Ten patients have good renal function.

## Conclusion

The number of patients with amyloidosis of FMF requiring RRT in Armenia is alarming. Prevention of RA by early diagnosis and early intervention (colchicine) must be intensified. Outcome of HD was poor in contrast to renal Tx. Results after renal Tx were much better than for HD and did not differ greatly from non-FMF pts, apart from drug interaction.

## Disclosure of interest

None declared

